# Nitric Oxide: Perspectives and Emerging Studies of a Well Known Cytotoxin

**DOI:** 10.3390/ijms11072715

**Published:** 2010-07-16

**Authors:** William A. Paradise, Benjamin J. Vesper, Ajay Goel, Joshua D. Waltonen, Kenneth W. Altman, G. Kenneth Haines, James A. Radosevich

**Affiliations:** 1 Center for Molecular Biology of Oral Diseases, College of Dentistry, University of Illinois at Chicago, Chicago, IL 60612, USA; E-Mails: paradise@uic.edu (W.A.P.); vesperbe@uic.edu (B.J.V.); 2 Department of Jesse Brown, Veterans Administration Medical Center, Chicago, IL 60612, USA; 3 Division of Gastroenterology, Department of Internal Medicine, Charles A. Sammons Cancer Center and Baylor Research Institute, Baylor University Medical Center, Dallas, TX 75246, USA; E-Mail: ajay.goel@baylorhealth.edu; 4 Department of Otolaryngology, Wake Forest University, Winston-Salem, NC 27157, USA; E-Mail: jwaltone@wfubmc.edu; 5 Mount Sinai School of Medicine, New York, NY 10029, USA; E-Mail: Kenneth.altman@mountsinai.org; 6 Department of Pathology, Yale University School of Medicine, New Haven, CT 06510, USA; E-Mail: k.haines@yale.edu

**Keywords:** nitric oxide, epigenetics, cytotoxicity, high NO adaptation, oncogenetic

## Abstract

The free radical nitric oxide (NO^•^) is known to play a dual role in human physiology and pathophysiology. At low levels, NO^•^ can protect cells; however, at higher levels, NO^•^ is a known cytotoxin, having been implicated in tumor angiogenesis and progression. While the majority of research devoted to understanding the role of NO^•^ in cancer has to date been tissue-specific, we herein review underlying commonalities of NO^•^ which may well exist among tumors arising from a variety of different sites. We also discuss the role of NO^•^ in human physiology and pathophysiology, including the very important relationship between NO^•^ and the glutathione-transferases, a class of protective enzymes involved in cellular protection. The emerging role of NO^•^ in three main areas of epigenetics—DNA methylation, microRNAs, and histone modifications—is then discussed. Finally, we describe the recent development of a model cell line system in which human tumor cell lines were adapted to high NO^•^ (HNO) levels. We anticipate that these HNO cell lines will serve as a useful tool in the ongoing efforts to better understand the role of NO^•^ in cancer.

## 1. Background

NO^•^ is a free radical which was discovered in 1980 as a ubiquitous diffusible second messenger. While some authors use the term “nitric oxide” to refer to any of the nitric oxide reactive species (NO^•^, NO^−^, and NO^+^), in biological settings, nitric oxide usually refers to the free radical. NO^•^ was determined to be a prominent constituent of what was then called endothelium-derived relaxing factor (EDRF) [1]. Later in 1985, *Eschericia coli* lipopolysacchride (LPS) was found to initiate production of NO^•^ by LPS-stimulated mouse macrophages [2]. We now know the molecule plays a significant role in both normal and abnormal physiology of human beings, as well as plants and invertebrates [3–5]. NO^•^ has a well characterized two-step synthetic metabolic pathway in which *L*-arginine is first converted to *N*_G_-hydroxy-*L*-arginine and then to *L*-citrulline and NO^•^. This reaction is catalyzed by the enzymatic family of nitric oxide synthases (NOSs). Three NOS isoforms exist: 1) nNOS/NOS1, a calcium-dependent enzyme discovered in neurons that is involved in neural transmission; 2) iNOS/NOS2, a calcium-independent enzyme that releases large amounts of NO^•^ in response to macrophage activation with endotoxin and cytokines, and is involved in cytotoxicity; and 3) eNOS/NOS3, also a calcium-dependent enzyme that is constitutively expressed, isolated from endothelial cells, and is found in normal vascular endothelium [6,7]. Once NO^•^ is produced, it can react with various molecules, resulting in more stable compounds such as S-nitrosothiols, metal adducts, peroxynitrites (while in the presence of oxygen), and tetrahydrobiopterin (THB) [6]. THB is recognized as a necessary prerequisite for the biosynthesis of key aromatic amino acid hydroxylase enzyme precursors necessary for synthesis of neurotransmitters such as serotonin, melatonin, epinephrine and dopamine.

It is believed NO^•^ regulates the physiology and pathophysiology of the body through one of three biomolecular mechanisms: 1) redox interactions with thiols, 2) coordinating interactions with metal functional centers, and 3) through protein kinase activity [8]. The role and impact of NO^•^ is believed to be widespread because of its ability to cross cell membranes in an unaltered chemical form, diffuse rapidly, interact with key generative and target cell-response proteins, and quickly interact with key transition metal containing proteins [6].

The various biomolecular mechanisms of NO^•^ result in numerous biological functions, including: 1) antitumor and microbial immunity, including against gram positive organisms [6], 2) immuno-modulation and allo-antigenicity [9,10], and 3) a signaling pathway [6]. Nearly every cell throughout the body has the ability to express calcium-independent iNOS [6]. Within the central nervous system, learning, sleep, feeding, male and female reproductive behavior are all impacted by NO^•^ [11]. It also influences the neurotransmitters in synapses between peripheral organs [11] and regulates angiogenesis and neurogenesis after stroke activity [12]. NO^•^ also delays the aging of oocytes [13], controls resting potential in skeletal muscle [14], regulates contraction-excitation coupling [14], and modulates chondrocyte development during endochondral ossification [15]. Significantly, NO^•^ depravation is a critical underlying cause for endothelial dysfunction, which in turn is a key common contributor to diabetes-related cardiovascular disease, myocardial infarction, and atherosclerosis [16,17].

NO^•^ in low concentrations is now known to have benign, modulating, and regulatory effects on normal mammalian and human biology and physiology. Higher concentration levels of NO^•^ are now shown to be both damaging and pathologic to physiologic processes [6]. What is defined as a high or low level can vary enormously depending upon which physiologic system the free radical is found and the apparent contradictory functional impact of NO^•^ presence on a particular molecular mechanism and biochemical microenvironment [6]. For instance, at NO^•^ concentrations of less than 100 nM, cyclic guanosine monophosphate (cGMP), cGMP-dependent protein kinase (PKG) and extracellular signal regulated kinase (ERK) activation can occur. As concentration levels increase Akt is phosphorylated, and in ranges between 300 to 800 nM hypoxia inducible factor-1 alpha and p53 are stabilized [18]. As concentrations increase further, nitrosation and oxidative processes become prominent and initiate stressful cellular events [19]. However, the role of NO^•^ can be either protective or toxic, depending upon the unique biochemical content of the microenvironment in which it exists [20].

It is also now well established that NO^•^ plays a multifaceted and contradictory role in the biology and growth of tumors [21]. Over-expression of NOS has been shown to be responsible for tumor angiogenesis and maintaining vascular tone within tumor blood vessel systems [22–25], as well as the facilitation of neoplastic transformation [22,26,27]. Studies have shown that in cancer patients, NO^•^ regulates blood flow to tumors, and by down-regulating NO^•^ synthesis, a distinct vasoconstricting event results [22]. This has been demonstrated through the use of *N*-nitro-*L*-arginine (*L*-NNA) to reduce blood flow to tumors in BD9 rats with P22 carcinosarcoma [22,28]. In humans cancer patients reducing NOS results in an increase in blood pressure [22,29,30]. At higher concentrations in the proper microenvironment, for an extended period of time, NO^•^ exposure initiates inflammation, can stimulate tumor growth and/or metastatic behavior [6,7], and can lead to mutations and the clinical presentation of cancer [9,31,32]. Exogenous sources of NO^•^, such as cigarette smoke, contribute to subcellular damage through the formation of *N*-nitrosoamines and N-nitrosamides, contributing to elevated expression of head and neck cancers [7,33–36].

It is also well recognized that reactive oxygen species (ROSs) play an important function in either the protective or pathologic expression of NO^•^ reactions with oxygen [6,7]. ROSs provide beneficial impacts through killing of microorganisms and malignant cells, or a pathologic effect, again depending upon the microenvironment. Higher concentrations of ROS generate oxidative and nitrosative environmental stressors leading to: 1) DNA damage, 2) down-regulated antioxidants, and 3) an impact on transcription/translation activities, thereby generally impairing normal cellular function [7,37,38]. Should DNA become damaged from either endogenous or exogenous sources of NO^•^, a number of defensive apoptotic systems are initiated to protect against unwanted cellular transformation [6,7]. Amongst the most important defensive apoptotic systems is the up regulation of DNA damage sensing proteins, such as p53. Damage not repaired typically results in cellular death through apoptosis. NO^•^ is also known to inhibit caspase activation, which in turn is known to induce normal apoptosis. Studies have also shown NO^•^ prohibits apoptosis in a variety of cell types, as well as in some tumor cells [39,40].

## 2. GST-pi

Thiols are a group of biological molecules which act as intracellular antioxidants. Among the most studied forms is glutathione (GSH), known to react with and neutralize electrophilic centers of a number of environmental and oxidative cellular stressors. The enzyme which catalyzes these reactions is glutathione S-transferase-pi (GST-pi), one of a family of Phase II detoxifying glutathione S-transferases (GSTs) responsible not only for detoxification, but also activation of significant biochemical pathways essential to normal physiology [41]. These oxidative stressors include elevated levels of NO^•^. The GST isoenzymes and their behavior are essential to providing yet another tool toward protection of DNA from a variety of endogenous and exogenous pathogenic sources [42–44]. Equally important, the GSTs catalyze the conjugation of GSH to an array of xenobiotic or toxic compounds rendering them non-toxic [45]. Due to these behaviors GSTs are an important area of research in molecular biology.

While GST isoenzymes relieve the source of toxicity, the catalytic group is also strongly implicated in the development of cellular resistance to anti-cancer drug therapy [42,45]. It provides a mechanism to explain cancer patients’ observed resistance to anticancer drug therapies [45]. Tumor cell lines which over-express GST-pi have heightened detoxification responses and acquire increased resistance to compounds perceived by the body as being toxic, including chemotherapeutic drugs [42,45,46]. GST isoenzymes are categorized into three primary groups: 1) cytosolic, 2) membrane-bound microsomal, and 3) mitochondrial [45]. The cytosolic type is further divided into seven classes: 1) Alpha, 2) Mu, 3) Omega, 4) Pi, 5) Sigma, 6) Theta, and 7) Zeta [45]. GST-pi is now recognized to be the predominant isoform subclass [42,44].

The GST-pi gene has four functional polymorphisms (GSTP1*A, GSTP1*B, GSTP1*C, and GSTP1*D), with each allelic genotype having different treatment response outcomes among individual cancer patients [45,47]. Examples include: 1) GSTP1*A is responsible for acquired resistance to cisplatin treatment due to creation of platinum-GSH conjugates [48], 2) GSTP1*B, which in certain circumstances, is associated with an impaired ability to detoxify platinum based therapeutic treatment [49], and 3) patients testing positive for GSTP1*C appear to experience breast cancer with less frequency [50]. All polymorphisms have been shown to impact, to varying degrees: 1) anticancer therapy treatment, 2) chemotherapeutic response, and 3) susceptibility to cancer. Most importantly, GSTP1 has been reported to be over-expressed in a number of different tumor types including: colon, lymphoma, pancreas, breast, NSCLC and ovarian [42,51]. One effort analyzed GST enzymes, GST composition, and GSH concentration levels in normal and squamous cell carcinoma tissues among 25 patients (14 with oropharyngeal or oral tumors, 11 with laryngeal tumors) [44]. GST-pi levels increased in 11 of the 14 oral cavity tumors, and elevated expression of GST-pi was found in all laryngeal tumors [44]. Another report provides evidence for heighten risk of relapse for laryngeal cancer associated with GST-pi over-expression [52]. Others propose the possibility that up-regulated GST-pi, GST-mu, and GST-alpha can be predictors of a second primary tumor in head and neck cancers [53]. Additional studies demonstrate over-expression of GST-pi within normal mucosa adjacent to tumors, dysplastic mucosal lesions, and head & neck squamous cell carcinoma (HNSCC) [54,55]. GST-pi expression increases through a step-wise progression, correlating with up-regulated NOS and molecular markers of oxidative injury [54,55]. It has been hypothesized that GST-pi is over-expressed in mucosal cells in response to oxidative injury by toxins such as NO^•^ and known nitrosative carcinogens resulting from smoking cigarettes [54,55].

Presented herein is a study in which we investigated GST-pi expression in laryngeal tumors (Table 1). Patient charts were reviewed for TNM stage and course of treatment. Tissue sections were reviewed, and the intensity of tumor staining was graded on an immunohistochemical scale (0–4).

**Figure 1.**
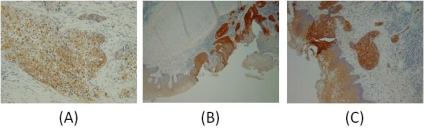
GST-pi immunostaining in human laryngeal tumors. (**A**) Patient 10, who had failed prior surgical treatment without radiation therapy; (**B**) Patient 5, who had failed prior treatment with radiation therapy; (**C**) Patient 7, who had failed previous treatment with radiation therapy. Positive immunohistochemical staining is brown. Images collected at 100× magnification.

In ten patients, seven had previously undergone radiation therapy; five of the seven patients were concurrently treated with chemotherapy and radiation therapy. All patients failed treatment or had recurrence or persistent disease. The seven patients who received radiation exhibited higher levels of GST-pi expression than the three patients who were not treated with radiation. Figure 1 shows examples of GST-pi immunostaining observed in human this study.

To investigate the commonality among squamous cell carcinomas arising in different sites, we also investigated the NOS and GST-pi expression of cervical squamous cell carcinomas (CSCC). Presented herein are results showing expression of eNOS, iNOS, and GST-pi in a series of patients with CSCC. Patient charts were reviewed for TNM stage, tumor grade, and course of treatment. All samples were obtained prior to treatment. Tissue sections were reviewed; the intensity of tumor staining was graded on an immunohistochemical scale (0–3). Table 2 summarizes the results.

**Figure 2.**
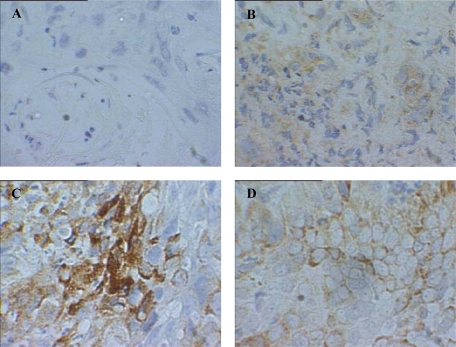
Immunohistochemical staining for (**A**) no primary antibody control; (**B**) eNOS, (**C**) iNOS, and (**D**) GST-pi in a single cervical sample. Positive immunohistochemical staining is brown. Strong staining is observed for iNOS and GST-pi, while little eNOS staining is apparent. Images collected at 250x magnification.

Both iNOS and GST-pi were highly expressed in CSCC, whereas eNOS showed only limited expression. Examples of the observed staining are shown in Figure 2. The eNOS expression in CSCC was in contrast to previously reported HNSCC work which showed highly expressed eNOS [56,57].

Another reported study confirms our findings that NO^•^ is a significant contributor to cervical cancer, and suggests a link between NO^•^ and a number of prominent risk factors associated with the onset of cervical cancer. These factors include: 1) chronic inflammation, 2) HPV infections, 3) extended use of oral contraceptives, 4) sexually transmitted diseases, and 5) smoking tobacco [58]. All of these factors cause increases in NO^•^ levels [58–61] and markers of NO^•^-mediated mutagenesis in patients with cervical intraepithelial neoplasia [58,62,63].

## 3. Reactive Nitrogen Species

The role of reactive nitrogen species (RNSs) has been well documented for many decades. The impact of RNSs originates in inflammatory tissues and can result in mutations in tumor suppressor genes, leading to subsequent tumor neoplastic growth [64]. RNSs also cause post-translational modifications of proteins involved in fundamental cellular functions such as apoptosis, cell cycle check point, and DNA repair [65]. RNSs are known to cause both oxidation and nitration reactions resulting in DNA strand breaks, mutations in DNA base pairs, and helix modifications [65]. What has also become increasingly evident over time is how important the molecular composition of the microenvironment is relative to the degree of DNA alterations. The molecular composition of the microenvironment is influenced by a number of RNS factors, including: 1) biomolecular profile, 2) type, 3) concentration level, 4) accessibility, 5) bioavailability, and 6) half life [65]. RNSs can evolve further into a variety of related molecules including 4-hydroxynoneal (4-HNE) and reactive aldehydes-malondialdehydes (MDA), both of which are associated with increased cancer risk in chronic inflammatory diseases [65–67]. Both 4-HNE and MDA are also known to cause point mutations within tumor suppressor genes [65–67]. Further, RNSs play a critical role as a facilitator between signal transduction receptors such as the MAPK signaling cascade. This can lead to the expression of proto-oncogenes such as c-Jun, c-Fos, and AP-1. These proto-oncogenes impact differentiation, proliferation, cellular death, and transformation [65,68,69]. Free radical exposure is also well recognized to cause post-translational modifications which affect the functionality of key cellular proteins. For example, exposure to NO^•^, an abundant RNS, leads to post-translational modifications of both p53 and Rb tumor suppressor genes at critical concentration levels [65,70,71]. Exposure to NO^•^ also activates DNA repair and signal transduction species including DNA protein kinases [65,72,73].

## 4. Epigenetics and NO^•^

In the early 1940’s C.H. Waddington first used “epigenetics” to describe the mechanisms responsible for the developmental pathway from fertilized egg to an adult [74–76]. Epigenetics is known to regulate primary biological functions, including, but not limited to: 1) memory function, 2) development and aging, 3) mobile elements activity, 4) genomic imprinting, 5) viral infections, 6) somatic gene therapy, 7) cloning, 8) X-inactivation, and 9) the biology of cancer [77–81]. The list of diseases associated with epigenetic dysregulation continues to grow as research efforts progress [77–81]. Over time and with the expanded knowledge base created by the efforts of many, the term has evolved to reference the heritable modifications to chromatin, which regulate gene expression, but do not change the underlying DNA sequence [75,78,82]. The impact on chromatin composition can be rapid and reversible, originating from endogenous and exogenous sources and which may well modulate gene expression behavior [47,74,75,82].

Gene expression and silencing can be carried out via a number of interrelated epigenetic mechanisms that may be modulated by NO^•^. These mechanisms include, but are not limited to: 1) DNA methylation [75,82–84], 2) microRNA (miRNA) [82,85,86], and 3) histone modifications [75,78,82,83,85,87–89]. The three mechanisms combine synergistically to regulate and affect epigenetic programming and reprogramming behavior [82,85,90–97]. Herein we discuss these three mechanisms and the emerging research to date as to how these may be affected by NO^•^. Interestingly, a recent study involving Duchenne muscular dystrophy indicated that a diminution of NO^•^ results in global epigenetic changes, thereby implicating NO^•^ as an “epigenetic molecule” [5].

### 4.1. DNA Methylation

Chromatin is made up of nucleosomes. The nucleosomes are comprised of DNA (146–147 base pairs in length, depending upon the literature cited [82,84]) and histones. The DNA is wrapped in a left-handed super-helix 1.7 times surrounding a core complex of eight histones [84], two each of H2A, H2B, H3, and H4 [82,86]. Each histone within the core has two active functional regions: 1) a “histone-fold” area to facilitate histone-to-histone and histone-to-DNA interactions in nucleosomes, and 2) a NH_2_-terminal with COOH-terminal “tails,” which are the sites for post-translational modifications that include methylation, phosphorylation, ubiquitination, and acetylation [84]. The tails also appear to facilitate linkage between other nucleosomes and/or DNA [87]. Chromatin also allows DNA molecules, comprised of millions of nucleotides, hundreds of millions of base pairs in length, to be housed highly compressed within the cell nucleus [82,98]. Less tightly bound chromatin usually has more reactive sites available for histone alterations, which in turn reversibly modify chromatin structure [82].

The DNA methylation and chromatin reconfiguration processes have equally prominent, yet reversible roles in mediating the genome into transcriptionally expressed or unexpressed segments [77,78]. Some patterns of DNA methylation remain constant throughout adulthood, while others are reversible. The on-going and mutable role of histones is reversible and also facilitates the silencing or unsilencing of gene expression. Tumorigenesis is a key example of pathological dysregulation in chromatin remodeling, or a lack of normalcy in DNA methylation processing behavior [77,78]. DNA methylation involves the addition of a methyl group at the carbon 5 position of the cytosine ring. The event is reversible and is a significant factor in gene expression [77,78]. It takes place primarily within the 5’CG3’ (also known as the CpG dinucleotide or CpG loci or sites), which are usually depleted and irregularly positioned throughout the genome with weakly concentrated locations. However, more dense areas known as CpG islands also exist [89,90,93,99]. CpG sites are usually methylated whereas the CpG islands are unmethylated. As we age, this mechanism reverses with intermittent methylation of the CpG islands taking place with a corresponding loss of overall methylation patterns throughout the genome; this is prominent with oncogenic events [82,90,99]. Abnormal or DNA hypermethylation patterns are known to impact promoter regions, which in turn, silence genes and are strongly evident in most cancers. Methylation anomalies also fail concurrently to express many tumor suppressor genes, further contributing to oncogenesis [82,90,91,99]. The specific relationship among CpG island hypermethylation activity, genetic alteration, and epigenetic inactivation of tumor suppressor genes is currently being studied in colorectal cancers (CRCs) [100]. CpG islands exist in about 50% of all human genes within promoter regions, and when hypermethylated, result in transcriptional silencing and tumor suppressor gene activity being down-regulated [100]. A smaller group of CRCs demonstrate extensive methylation behavior referred to as CpG island methylator phenotype (CIMP). There are three principle mechanisms driving genomic instability in CRCs: 1) CIMP, 2) microsatellite instability (MSI), a unique phenotype within CRC, and 3) chromosomal instability (CIN). All three mechanisms all contribute to epigenetically alter gene expression in CRCs [101,102].

DNA methylation is facilitated by DNA methyltransferases (DNMTs), including DNMT 1, DNMT 3A, and DNMT 3B [92,99]. Collectively, all three enzymes ensure proper DNA methylation patterns [95,99]. DNA methylation (see Table 3 below) is being studied because cancer cells display elevated levels of altered DNA methylation patterns when compared to normal cells [77,78]. There are a variety of tumor types with associated hypermethylation of at least one gene, including: lung cancer, breast cancer, leukemia, and hematologic diseases [77,78,94,96,97]. Certain patterns of hypomethylation can also contribute to the formation of other cancer types as well, including but not limited to: metastatic hepatocellular cancer, cervical cancer, prostate and B-cell chronic lymphocytic leukemia [77,78,103–106]. It has been reported that transcription is impeded when methylcytosine binding domain proteins (MBDs) bind to methylated DNA. This binding process interferes with the interdependent relationship between the DNA methylation and chromatin reconfiguration processes, thereby precluding further gene transcription [78,90].

An additional study demonstrated that NO^•^ regulates chromatin and gene expression through inactivation of nuclear and cytoplasmic proteins by tyrosine-(Tyr) nitration and/or *S*-nitrosylation/nitrosation [133]. In *S*-nitrosylation, primary, tertiary and quaternary protein architecture is affected [5]; in Tyr-nitration, the impact is more widespread across a variety of proteins [38]. Although NO^•^ is diffusible, it must also exist physically within the proper microenvironment and limited macroenvironment with proteins/substrates in order to react. Evidence of this includes the presence of iNOS in the caveolae of endothelial cells, in neurons, and within the nucleus [5,134]. Additionally, it has been suggested that NO^•^, through S-nitrosylation, impacts a number of targets: 1) a variety of transcription factors, including tissue specific transcription factors, 2) some oncoproteins, 3) DNA binding, and 4) transactivation of nuclear receptors [5]. NO^•^-mediated changes on transcription factors through Tyr-nitration have also been reported; these changes primarily through impacting normal protein-to-protein interactions and limiting nuclear localization [5]. Table 4 lists the impact of NO^•^ on various epigenetic modulators.

It has further been suggested that DNA is susceptible to transition metal-mediated reductive/oxidative modifications which can affect double helix strength [5,135]. Others have hypothesized NO^•^ may be impacting a “genome-wide oscillation” and expression/suppression of hundreds of genes, through reactions with thiols/cystine residues in partnership with Fe^2+^ ions (both of which are believed to reside in chromatin) [5,136,137]. This “genome-wide oscillation” could remain in place with oscillating cycles, constructing and deconstructing—of protein complexes, resulting in a corresponding impact on transcription processing [5,138]. Finally, there is evidence to suggest synthesis of NO^•^ within the nucleus due to the presence of THB enzymes and two isoforms of NOS (eNOS and iNOS) [5,139].

### 4.2. MicroRNA

MicroRNAs are relatively small, non-coding RNAs, typically 20–23 nucleotides in size. miRNAs originate from 60–110 nucleotide fold-back RNA precursors [90] and have an enormous impact on the control of gene expression [99,140,141]. The biosynthetic pathway originates from proteins of the Argonaute family. These proteins are transcribed first via RNA polymerase II, and then by RNases III Drosha and DGCR8. Finally, in the cytoplasm, RNA III Dicer transforms the proteins into the fully functioning miRNA [99,140]. Typically miRNAs act as post-transcriptional regulators by impeding protein production of specific messenger RNA (mRNA) molecular species and apparently interacting through base-pairing between the 5’-end tails of miRNA (nucleotides 2–8) and the anti-parallel sequences of the 3’-untranslated (3’-UTRs) areas within selected mRNAs [141–144]. Originally believed to be relatively small in number, there are now over 460 human miRNAs identified [99], with the possibility of greater than 1,000 in existence [140]. They have been linked to aberrant cell growth patterns and appear to play a dual role in both oncogenesis and tumor suppression, depending upon which portion of the genome is being affected. For example, the miR-17-92 cluster has a role in tumor neovascularization, when c-myc activates transcription [145,146]. This gene is found to be over-expressed in the miR17-92 cluster in B-cell lymphomas and lung cancers. c-Myc also contributes to tumor angiogenesis, as well as tumor growth in mouse B-cell lymphoma [145–148]. It has also been demonstrated that human cancer types can be classified using expression patterns of miRNA [149]. Furthermore, at least one significant relational link has been identified between DNA methylation patterns and miRNA; the strength of this relationship is possibly affected by changing the amounts of DNMT1, DNMT 3A, and/or DNMT 3B present [98,150,151].

As is the case with DNA hypermethylation, regulating the abnormal activity levels of certain miRNAs could be important in initiating and controlling tumorigenesis. It may be possible to target these species through interventions or blocking drugs, such that the miRNAs serve as therapeutic molecular markers. Increasing efforts are also being directed towards providing evidence to support the development of miRNAs as diagnostic and prognostic products [140]. A few examples include: 1) stimulating apoptosis in cultured glioblastoma cells by deprogramming miR-21 (an oncogenic miRNA) [152,153], 2) up-regulating miR-372 and 373 in testicular germ cell tumors [99,154], and 3) over-expressing miR-155 in both breast cancers and B-cell lymphomas [99,155–157]. Initial clinical results using epigenetic drugs, such as 4-phenylbutyric acid (PBA) and 5-Aza-2’-deoxycytidine (5-Aza-CdR) exhibit the capability to up-regulate miR-127, which in turn down-regulates Bcl6. This finding provides hope that additional therapeutic options will become available by developing drugs that act via epigenetic mechanisms [99,158]. It also suggests that epigenetic drugs may be able to up-regulate tumor-suppressor genes abnormally de-programmed epigenetically, while also causing miRNAs to turn off oncogenic mRNAs [99,159].

As research on miRNAs continues to emerge, a number of studies have found a link between NO^•^ expression and a number of different miRNAs. In one study, human umbilical vein endothelial cells (HUVECs) were exposed to prolonged unidirectional shear stress, which resulted in the significant up-regulation of 13 miRNAs [160]. Among the 13 miRNAs identified, miR-21 exhibited the greatest level of up-regulation. miR-21 serves as a regulator of smooth muscle apoptosis [161] and has been found to be regulated in both cardiac hypertrophy [162] and human tumors [163]. Notably, HUVECs which over-expressed miR-21 exhibited increased eNOS phosphorylation and NO^•^ production, as well as decreased apoptosis. Similarly, another study found that decreasing the levels of miR-145—another smooth muscle miRNA regulator—resulted in decreased NO^•^ expression [164].

Two other miRNAs have been shown to indirectly modulate iNOS expression: miR-155 and miR-661. Mice transfected with miR-155 exhibited reduced expression of Suppressor of Cytokine Signal-1, and in turn, enhanced iNOS expression [165]. In a different study, human liver cancer cells expressing the hepatitis B virus transactivator protein HBx were studied [166]. When the miRNA miR-661 was depleted in these HBx-expressing cells, HBx activity was impaired, leading to enhanced iNOS and nitrite production.

### 4.3. Histone Modifications

Histones are yet another fundamental epigenetic pathway mechanism and are influential in both transcriptional and post-translational modifications [5,82]. These proteins are positively attracted to the more negatively charged DNA molecules present, making them particularly susceptible to post-transcriptional changes in DNA binding through: 1) acetylation, 2) methylation, 3) phosphorylation, 4) ubiquitination, 5) SUMOylation (small ubiquinine-like modifier), and 6) isomerization [5,78]. More specifically, they also include: 1) lysine acetylation, 2) lysine and arginine methylation, 3) serine and threoine phosphorylation, 4) lysine ubiquitylation, and 5) lysine SUMOylation, with over 60 modification sites currently known [82]. Histone post-translational modifications occur in the globular domains and the amino-terminal tails [82,167,168], and along with ATP–dependent chromatin remodeling, are among the most significant influencers of gene expression [82]. Coupled with DNA methylation activities, these histone mechanisms collectively create an adaptive epigenetic environment [82,169]. This is of particular importance towards understanding the enormous impact the histone post-translation changes can have on chromatin steric formation. By altering the molecular landscape, it transforms transcriptional regulators to interact with *cis*-DNA binding elements [82]. This pattern has been studied and verified in lysine acetylation [82,170].

Histone modification activities take place primarily through two groups of enzymes. The first, histone acetyltranferases (HATs) is comprised of three classes: GNAT, MYST, and CBP/p300. They are characterized by the ability to transfer acetyl groups from acetyl-CoA to amino-ɛ groups for lysines within H3 and H4 and are principally responsible for the opening up of chromatin structure, thereby permitting access for transcription processes to take place [171]. The second group, histone deacetylases (HADCs) reverse the process, resulting in a tightening or constriction of chromatin, making the epigenome less accessible to reactions [172]. There are four classes of HDAC enzymes [82]. Class I consists of HDAC1, HDAC2, HDAC3, and HDAC8. Class II is further divided into two subgroups: IIa, which includes HDAC4, HDAC5, HDAC6, and HDAC7; and IIb, which includes HDAC9 and HDAC10. Class III consists of the sirtuins (SIRT1, SIRT2, SIRT3, SIRT4, SIRT5, SIRT6, and SIRT7) [173,174]. Class IV is comprised of one enzyme, HDAC11 [172,174]. All play significant roles in human physiology and processes, ranging from embryogenesis and cellular differentiation to tumorigenesis, by facilitating deacetylating enzymatic reactions [175–177]. Significantly, HATs are drawn to promoter locations to become part of protein complexes, and many transcriptional co-activators demonstrate HAT enzymatic characteristics [82]. Co-activators have emerged as a significant participant in chemical signaling between systemic and cellular metabolism, including regulating both mitochondrial oxidative metabolism and the balance between lipid, glucose, and energy homeostatic functionality [178]. There are a number of other histone modification pathways which we will not explore within the limitations of this review including, but not limited to: 1) histone methyltransferases, 2) lysine and arginine methyltransferases, and 3) non-SET K-methyltransferases. There is synergy and transience between the HATs and HDACs which creates an oscillating-feedback loop and reversible gene transcription control mechanism environment. Table 5 indicates some of the cancers believed attributable to dysregulated histone protein behavior within the epigenome.

The most recent advances in epigenetic research have been aided by a concurrent evolution of many laboratory techniques, including: 1) cDNA microarray, 2) restriction landmark genomic scanning, 3) CpG island microarrays, and 4) sodium bisulfate conversion [77,78,179]. Sodium bisulfate is particularly useful in differentiating areas of normal and abnormal methylation activity by converting unmethylated cytosines to uracil, while leaving methylated cytosines intact. There are a number of methods useful in exploring CpG island methylation patterns, including: 1) combined bisulfate restriction analyses, 2) methylation-sensitive single nucleotide extension, 3) methylation-sensitive single-strand conformational polymorphism, and 4) methylation-specific polymerase chain reaction assays [77,78,179,180]. Research efforts have also indicated that preemptive assessment of methylation patterns can predict possible malignancies and aid in more timely detection and diagnosis of tumors [77,78].

While much progress has already been made, it is clear that the analytical techniques used to study epigenetics are still evolving. For example, researchers have attempted to analytically differentiate between DNA cytosine methylation (5mC), and hydroxymethylcytosine (hmC). 5mC is involved with transcriptional repression, while the functionality of hmC is still unknown. A recent report concludes the two compounds are “experimentally indistinguishable” from one another using established 5mC mapping criteria and suggest existing 5mC data-bases should be re-examined to ensure that no hmC data has been included erroneously [181].

To date, only limited data exists regarding the role of NO^•^ in histone modification. A recent study has shown that eNOS gene expression relies upon underlying epigenetic causal mechanisms [82]. It was found that when the human eNOS gene in vascular endothelial cells is expressed, the promoter region is free of DNA methylation, and histone complexes initiate post-transcriptional changes. H3 lysine 4 methylation, H3 lysine 9 acetylation, and H4 lysine 12 acetylation all impacted chromatin by inducing an open steric formation. These reactions thereby permit access by appropriate transcription factors and mechanisms, most important among them, RNA polymerase II at the eNOS promoter location [82]. In contrast, the iNOS gene was found to be silent in cultured endothelial cells containing hypermethylated CpG dinucleotides within the promoter while they are complexed with the methyl-binding protein, MeCP2. These reactions result in silencing of post-translational histones H3 lysine 9 methylation and are suggested to be prevalent in transcriptionally unexpressed heterochromatin. Sterically, the chromatin structure is tightly configured, and RNA polymerase II is not present [82]. It is also postulated that this and other studies provide evidence that non-expressing cells have the necessary transcriptional mechanisms to directly affect eNOS expression, and more significantly, a chromatin-linked down-regulating system which prevents eNOS from being expressed in non-endothelial cells [82,182–184].

A different study found that the hyporesponsiveness of the iNOS promoter in humans is at least partially due to epigenetic silencing in direct response to the hypermethylation of CpG dinucleotides and histone H3 lysine 9 methylation [168]. More specifically, the study found that the iNOS promoter was highly methylated at CpG dinucleotides in various human endothelial cells and vascular smooth muscle cells, two cell types in which iNOS induction is known to be difficult. Furthermore, a human pulmonary adenocarcinoma cell line (A549), a colon adenocarcinoma cell line (DLD-1), and primary hepatocyte cell cultures are all capable of iNOS induction [168]. The iNOS promoter is hypomethylated in DLD-1 cells that have been treated with a DNA methyltransferase. This stimulates both global and iNOS promoter DNA hypomethylation. Use of a chromatin immunoprecipitation assay showed significant presence of methyl-CpG-binding transcriptional repressor MeCP2 within the iNOS promoter location in these endothelial cells. In its entirety the study provided a definition of chromatin-based epigenetic mechanisms controlling human iNOS gene expression [168].

## 5. Biological Model System

As indicated earlier in this review, our prior work has focused on the role of NO^•^ in both squamous cell carcinomas (head & neck, cervix) and adenocarcinomas (lung, breast). We and others have reported a spectrum of NOS expression in patient populations of these tumors, as well as other tumor types. It has also been observed that patients who present with and/or progress to high levels of NOS expression portend to have poorer clinical outcomes than those with low level expression. It has been hypothesized that immune system cells are being killed by the comparatively high free radical NO^•^ environment encountered in the tumor bed [185]. Since there is no practical way to study this in human patients, we sought to produce a unique, *in vitro* tissue culture model system of free radical stressed tumor cells to determine if in fact they could adapt to increasing levels of NO^•^. The resulting model system would mimic the spectrum of NO^•^ expression found clinically [186].

Our model system was developed by “adapting” low NO^•^ expressing cell lines to increasing levels of NO^•^ donor. These “parent” cells were gradually exposed to high NO^•^ (HNO) levels, resulting in a new set of HNO cell lines. DETA-NONOate was selected as the NO^•^ donor for the adaptation process due to: a) its high level of free radical donation (two moles of NO^•^ per mole of DETA-NONOate), and relatively long half-life (approximately 24 h. at 37 °C and pH 7.4). During the adaptation process, the cell lines successfully withstood incremental increases of 25 μM DETA-NONOate. For each cell line, the adaptation endpoint was selected as the concentration in which the exogenous NO^•^ introduced to the cells was lethal to the parent cell lines. At this endpoint concentration, the HNO cells still grow robustly and are not morphologically altered from the original (untreated) parent cells. Six different parent/HNO cell line pairs have already been developed: one human lung adenocarcinoma cell line (A549) [186], one mouse lung adenocarcinoma (LP07) [186], and four human breast adenocarcinomas (T-47D, Hs578t, BT-20, and MCF-7) [187]. Ongoing work is focusing on extending this model to human head & neck, colon, prostate, and liver tumor cell lines.

**Figure 3.**
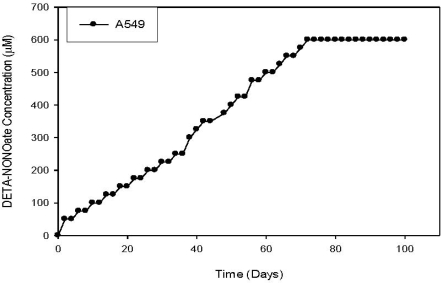
Adaptation of A549 human lung adenocarcinoma cell line to high nitric oxide (HNO) levels. Adapted from reference [186].

While the A549 cells were adapted to DETA-NONOate (see Figure 3 below), the A549-HNO cell lines were also found to be resistant to other nitrogen-based free radical donors [186,187]. This suggests the A549-HNO cell line could have been generated by using any appropriate NO^•^ donor, and that the cells were adapted to the NO^•^ free radical, and not the donor per se [186,187].

**Figure 4.**
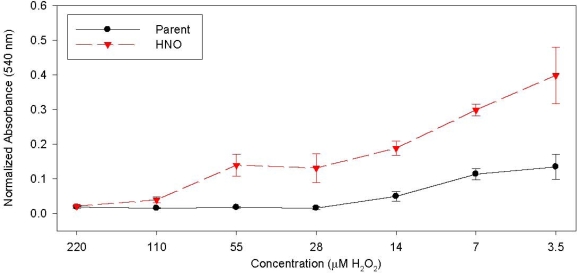
Treatment of T-47D cell lines (Parent and HNO) to varying concentrations of H_2_O_2_. Adapted from reference [187].

Additionally, the lung and breast tumor HNO cell lines were exposed to various concentrations of hydrogen peroxide (H_2_O_2_), an oxygen-based free radical donor [186,187]. The HNO cell lines were more resistant to exposure than the corresponding parent cell lines (see Figure 4 as an example). These results show that the HNO cells are similarly resistant to oxygen-based free radicals.

The reported adaptation process resulted in major biological changes, between the parent and HNO cells despite the identical morphology between the two. HNO cancer cell lines exhibited more aggressive growth than did their corresponding parent cell lines under both normal and low-nutrient growth conditions [186,187].

The HNO adapted cell lines are comparable to aggressive, fast growing tumors growing in high NO^•^ environments, while the parent cell lines represent less aggressive, slower growing tumors existing in relatively lower NO^•^ environments. Furthermore, our adaptation process demonstrated that long-term NO^•^ exposure can alter slow growing, less resistant tumors, into faster growing and more resistant cancer cells [186,187]. The molecular mechanism for this parent-to-HNO transformation remains to be elucidated; however, high concentration levels of NO^•^ (above 1 μM) are known to increase nitrosative cellular stress, which interferes with DNA repair and inhibits zinc finger complexes [187–189]. Our model system has proven that tumor cells are able to adapt to comparatively high NO^•^ concentrations, regardless of tumor origin or their histological type. Understanding the role of NO^•^ in tumor cells may in part lie with NO^•^-mediated epigenetics.

**Figure 5.**
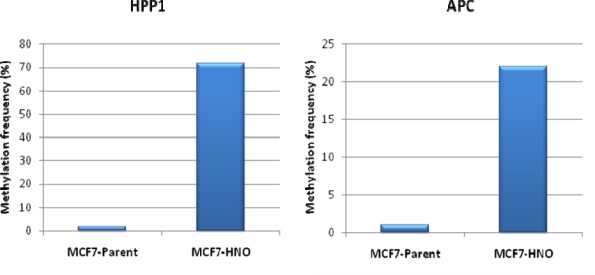
Methylation of HHP1 and APC in parent and HNO MCF-7 cells.

As was discussed above, epigenetic alterations that involve aberrant DNA methylation of CpG sequences in genes is increasingly being recognized as a key mechanism involved in transcriptional silencing of genes in both disease states and, healthy ageing populations [65,82,190]. Our HNO-adapted cell line system provides a robust, *in vitro* model for the identification of novel genetic targets that are associated with antioxidant stress. We also have evidence that, relative to the MCF-7 breast cancer parent cells, the HNO adapted MCF-7 cells demonstrate a significant increase in hypermethylation of both HPP1 (70-fold increase) and APC (22-fold increase) tumor suppressor genes (see Figure 5 below).

## 6. Conclusion

Research of NO^•^ has evolved greatly over time. The protective/cytotoxic duality of NO^•^, once in question, is now generally accepted. As such, current studies are now more intently focused on understanding the role of NO^•^ in cellular toxicity, particularly as it relates to tumor development and progression. The association between NOS and GST may be a key component of this story, given that over-expression of NOS (regardless of isotype) is often observed in parallel with the over-expression of GST (particularly GST-pi). As discussed above, these results are consistent across a number of different tumor types, suggesting that NO^•^ behavior may be more consistent across different tumor types than originally thought. Whether this commonality in NO^•^ behavior exists among different tumor types will become more apparent as research is further pursued in the field. The HNO cell line model system described above may prove to be a valuable tool for such studies. Similarly, work will continue in the area of NO^•^ and epigenetics. While epigenetic research is still in its infancy, it is already clear that NO^•^ may play an important role in a number of epigenetic functions, including DNA methylation, microRNAs, and histone modifications. Thus, while much is already known about the biological role of NO^•^, even more has yet to be discovered.

## Figures and Tables

**Table 1.** GST-pi immunohistochemical staining in human laryngeal tumors.

**Patient**	**Age/Sex**	**Tumor Location**	**Tumor Stage**	**Surgery Performed**	**Previous Treatment**	**GST-pi Intensity**	**GST-pi Pattern**
1	74/M	Larynx	T3N0M0 (recurrent)	Total laryngectomy	Chemo/XRT	3+	diffuse
2	79/F	Pyriform sinus	T3N0M0	Laryngopharyngectomy	None	1+	focal
3	73/M	Subglottis	T2N0M0	Total laryngectomy	XRT	3+	diffuse
4	75/F	Glottis	T4N0M0	Laryngopharyngectomy	None	1+	diffuse
5	73/M	Supraglottis	T2N0M0 (recurrent)	Supraglottic laryngectomy	XRT	4+	diffuse
6	63/M	Supraglottis	T4N2M0 (recurrent)	Completion laryngectomy	Supraglottic laryngectomy, Chemo/XRT	2+	diffuse
7	61/M	Supraglottis	T2N2M0 (recurrent)	Completion laryngectomy	Supraglottic laryngectomy, Chemo/XRT	4+	diffuse
8	51/M	Supraglottis	T4N0M0 (recurrent)	Laryngopharyngectomy	Chemo/XRT	3+	focal
9	77/M	Larynx	Recurrent	Total laryngectomy	Chemo/XRT	3+	diffuse
10	81/M	Larynx	T3N0M0 (recurrent)	Completion laryngectomy	Supraglottic laryngectomy	2+	focal

Chemo: chemotherapy, XRT: radiation therapy. Study was carried out with IRB approval.

**Table 2.** Cervical cancer patient summary and immunohistochemistry data.

**Patient**	**Age**	**Stage**	**Grade**	**Treatment**	**Recurrence/Persistence**	**DFS (mos.)**	**iNOS Intensity**	**eNOS Intensity**	**GST-pi Intensity**
1	39	IIB	2	C-R	N	32.5	3+	0	1+
2	48	IV	2	N/A	N/A	N/A	2+	0	1+
3	65	IIIB	3	C-R	Y	12.5	3+	0	0
4	41	IIIB	2	C-R	Y	4	2+	0	1+
5	39	IIIB	3	C-R	N/A	N/A	2+	1+	2+
6	50	IB2	3	C-R	N	37	3+	2+	2+
7	38	IB1	2	S	N	39	3+	1+	1+
8	49	IB2	2	C-R	N	38	3+	1+	2+
9	63	IIIB	2	C-R	Y	9	1+	0	1+
10	29	IIB	2	R	N/A	N/A	2+	0	1+
11	49	IIIB	2	C-R	Y	8	2+	2+	1+
12	49	IIB	3	C-R	N/A	N/A	2+	0	2+
13	61	IIIB	3	C-R	N/A	N/A	2+	1+	2+
14	63	IIB	2	C-R	N	40	3+	2+	2+
15	44	IIB	2	C-R	N	42	3+	1+	2+
16	44	N/A	2	N/A	N/A	N/A	2+	1+	1+
17	52	IVA	3	N/A	N/A	N/A	3+	1+	2+
18	39	IIB	2	C-R	N	39	2+	1+	2+
19	51	IIIB	2	C-R	N/A	N/A	2+	1+	1+
20	37	IB1	2	S-R	N	35	2+	0	1+
21	54	IB2	3	C-R	N	50	2+	1+	1+
22	49	IIA	3	R	N	49	2+	1+	1+
23	48	IIB	2	C-R	N	48	2+	0	2+

Staging by AJCC 2002 criteria. Treatment methods, C: chemotherapy, R: radiation therapy, S: surgery. DFS: Disease free survival. iNOS: inducible nitric oxide synthase; eNOS: endothelial constitutive nitric oxide synthase; GST-pi: glutathione S-transferase pi. Study was carried out with IRB approval.

**Table 3.** The epigenetic impact of dysregulated DNA methylation on gene expression and human cancers.

**Gene**	**Role/Function**	**Tumor Type/Location**	**Impact**	**Reference(s)**
APC	Inhibitor of β-catenin	Aerodigestive tract, lung, breast	Activation β –catenin route	[90,107–109]
AR	Androgen receptor	Prostate	Hormone insensitivity	[90]
BRCA1	DNA repair, transcription	Breast, ovarian	Double strand breaks	[90,110,111]
CDH1	E cadherin, cell adhesion	Breast, stomach, Leukemia	Dissemination	[90]
CDH13	H cadherin, cell division	Breast, lung	Dissemination	[90]
CDKN2A/p16	Cyclin-dependent kinase inhibitor	Head, neck, gastrointestinal tract, lung, NHL	Entrance in cell cycle	[78,90,108,112,113]
COX2	Cycloxyenase-2	Colon, stomach	Anti-inflammatory resistance^1^	[90]
CPBP1	Retinol-binding protein	Colon, stomach, lymphoma	Vitamin insensitivity	[90]
DAPK1	Pro-apoptotic	Lymphoma, lung, colon	Resistance to apoptosis	[90,108]
DKK1	Extracellular Wnt inhibitor	Colon	Activation Wnt signaling	[90]
DNMT1	DNA disruption	Various	Over-expression	[90]
DNMT3b	DNA disruption	Various	Over-expression	[90]
E-cadherin	Increasing proliferation, invasion and/or metastasis	Breast, Thyroid, Gastric		[114–116]
ER	Oestrogen receptor	Breast, prostate	Hormone insensitivity	[117,118]
EXT1	Heparan intermediate filament	Leukemia, skin	Cellular detachment	[90]
FAT	Cadherin, tumor suppressor	Colon	Dissemination	[90]
GATA4	Transcription factor	Colon, stomach	Silencing of target genes	[90]
GATA5	Transcription factor	Colon, stomach	Silencing of target genes	[90]
GSTP1	Conjugation to glutathione	Prostate, breast, kidney	Adduct accumulation	[90,108,119]
HIC1	Transcription factor	Various forms	Currently unknown	[90]
HOXA9	Homeobox protein	Neuroblastoma	Currently unknown	[90]
hMLH1	Defective DNA mismatch repair, gene mutations	Colon, Renal, Gastric, Endometrim, Ovarian		[116,120–122]
ID4	Transcription factor	Leukemia, stomach	Currently unknown	[90]
IGFBP3	Growth factor binding protein	Lung, skin	Resistance to apoptosis	[90]
Lamin A/C	Nuclear intermediate filament	Lymphoma, leukemia	Currently unknown	[90]
LKB1/STK11	Serine-theronine kinase	Colon, breast, lung	Currently unknown	[90]
MBD1	Rare mutations	Various	Over-expression	[90]
MBD2	Rare mutations	Various	Over-expression	[90]
MBD3	Rare mutations	Various	Over-expression	[90]
MBD4	Rare mutations	Various	Over-expression	[90]
MeCP2	Rare mutations	Various	Over-expression	[90]
MGMT	DNA repair of 06-alkyl-guanine, p53	Lung, brain, various	Mutations, chemosensitivity	[90,123,124]
MLH1	DNA mismatch repair	Colon, endometrium, stomach, ovarian	Frameshift mutations, gene mutations	[90]
NORE1A	Ras effector homologue	Lung	Currently unknown	[90]
p14^ARF^	MDM2 inhibitor	Colon, stomach. kidney	Degradation of p53	[90]
p15		Leukemia, Lymphoma	Entrance in cell cycle	[125–127]
p15^INK4b^	Cyclin-dependent kinase inhibitor	Leukemia, lymphoma, lung, SCC	Entrance in cell cycle	[90]
p16^INK4a^	Cyclin-dependent kinase inhibitor	Various	Entrance in cell cycle	[90,108]
p73	P53 homologue	Lymphoma	Currently unknown	[90]
PR	Progestrogen receptor	Breast	Hormone insensitivity	[90]
PRLR	Prolactin receptor	Breast	Hormone insensitivity	[90]
RARβ2	Retinoic acid receptor –β2	Colon, lung, head and neck	Vitamin insensitivity	[90]
RASSF1A	Ras effector homologue	Lung, breast, ovarian, kidney, nasopharyngeal	Currently unknown	[128–130]
Rb	Cell-cycle inhibitor	Retinoblastoma, oligodenodroglioma	Entrance to cell	[90,131,132]
RIZ1	Histone/protein methyltransferase	Breast, liver	Abnormal gene expression	[90]
SFRP1	Secreted frizzled-related protein 1	Colon	Activation Wnt signaling	[90]
SLC5A8	Sodium transporter	Glioma, colon	Currently unknown	[90]
SOC1	Inhibitor of JAK-STAT pathway	Liver, mieloma	JAK2 activation	[90]
SOC3	Inhibitor of JAK-STAT pathway	Lung	JAK2 activation	[90]
SRBC	BRCA1-binding protein	Breast, lung	Currently unknown	[90]
SYK	Tyrosine kinase	Breast	Currently unknown	[90]
THBS1	Thrombospondin-1, anti-angiogenic	Giloma	Neo-vascularization	[90]
TMS1	Pro-apoptotic	Breast	Resistance to apoptosis	[90]
TPEF/HPP1	Transmembrane protein	Colon, bladder	Currently unknown	[90]
TSHR	Thyroid-stimulating hormone receptor	Thyroid	Hormone insensitivity	[90]
VHL	Ubiquitin ligase component	Kidney, haemangioblastoma	Loss of hypoxic response	[90,129]
WIF1	Wnt inhibitor factor	Colon, lung	Activation Wnt signaling	[90]
WRN	DNA repair	Colon, stomach, sarcoma	DNA breakage, chemosensitivity	[90]

Abbreviations: NHL= Non-Hodgkin’s lymphoma, SCC= Squamous Cell Carcinoma, hMLH1= mutant homologue 1.

**Table 4.** The epigenetic impact of NO^•^.

**Substrate**	**Modification**	**Effect on Nucleosome/Chromatin**	**Transcription**
AP-1	S-N	Indirect	-
AtMYB2	S-N	Indirect	-
Class II HDACs	Dephosphorylation	Indirect	_
c-Myb	S-N	Indirect	-
GR	T-N	Indirect	+
HDAC2	S-N, T-N	Indirect	+
HIF-1α	S-N	Indirect	+
Histones	T-N	Direct	?
ikBα	T-N	Indirect	+
NF-kB	S-N, T-N	Indirect	-
Notch	T-N	Indirect	-
Nuclear receptors	S-N	Indirect	-
OxyR and SoxR	S-N	Indirect	+
P53	T-N	Indirect	-
PPARγ	T-N	Indirect	-
β –catenin	T-N	Indirect	-

Abbreviations: S-N= S-Nitrosylation, T-N= Tyr-Nitration. Adapted from reference [5].

**Table 5.** The epigenetic impact of histone modification on gene expression and human cancers.

**Gene**	**Tumor Type/Location**	**Impact**
CBP^1^	Colon, stomach, endometrium, lung, leukemia	Mutations, translocations, deletions
EZH2^3^	Various types	Gene amplification, over-expression
GASC1^4^	Squamous cell carcinoma	Gene amplification
HDAC1^2^	Various types	Aberrant expression
HDAC2^2^	Various types	Aberrant expression, mutations in MSI+
MLL1^3^	Haematological malignancies	Translocation
MLL2^3^	Glioma, pancreas	Gene amplification
MLL3^3^	Leukemia	Deletion
MORF^1^	Haematological malignancies, leiomyomata	Translocations
MOZ^1^	Haematological malignancies	Translocations
NSD1^3^	Leukemia	Translocation
p300^1^	Colon, stomach, endometrium	Mutations in MSI+
pCAF^1^	Colon	Rare mutations
RIZ1^3^	Various types	CpG-island hypermethylation

Abbreviations: MSI+= Microsatellite instable tumors. Footnotes:

1 Histone acetyltransferases,

2 Histone deactylases,

3 Histone methyltransferases,

4 Histone demethylase. Adapted from references [90,107,109].
